# Safe-by-Design Antibacterial Peroxide-Substituted Biomimetic Apatites: Proof of Concept in Tropical Dentistry

**DOI:** 10.3390/jfb13030144

**Published:** 2022-09-07

**Authors:** Ika Dewi Ana, Any Lestari, Prescillia Lagarrigue, Jérémy Soulie, Rahmi Anggraeni, Françoise Maube-Bosc, Carole Thouron, Benjamin Duployer, Christophe Tenailleau, Christophe Drouet

**Affiliations:** 1 Department of Dental Biomedical Sciences, Faculty of Dentistry, Universitas Gadjah Mada, Yogyakarta 55281, Indonesia; 2 Research Division, PT Swayasa Prakarsa, UGM Science Techno Campus, Yogyakarta 55571, Indonesia; 3 CIRIMAT, Université de Toulouse, CNRS, 31030 Toulouse, France

**Keywords:** peroxide, silver, biomimetic apatite, tropical dentistry, antibacterial properties, biocompatibility, porous scaffolds

## Abstract

Bone infections are a key health challenge with dramatic consequences for affected patients. In dentistry, periodontitis is a medically compromised condition for efficient dental care and bone grafting, the success of which depends on whether the surgical site is infected or not. Present treatments involve antibiotics associated with massive bacterial resistance effects, urging for the development of alternative antibacterial strategies. In this work, we established a safe-by-design bone substitute approach by combining bone-like apatite to peroxide ions close to natural in vivo oxygenated species aimed at fighting pathogens. In parallel, bone-like apatites doped with Ag^+^ or co-doped Ag^+^/peroxide were also prepared for comparative purposes. The compounds were thoroughly characterized by chemical titrations, FTIR, XRD, SEM, and EDX analyses. All doped apatites demonstrated significant antibacterial properties toward four major pathogenic bacteria involved in periodontitis and bone infection, namely *Porphyromonas gingivalis* (*P. gingivalis*), *Aggregatibacter actinomycetemcomitans* (*A. actinomycetemcomitans*), *Fusobacterium nucleatum* (*F. nucleatum*), and *S. aureus*. By way of complementary tests to assess protein adsorption, osteoblast cell adhesion, viability and IC_50_ values, the samples were also shown to be highly biocompatible. In particular, peroxidated apatite was the safest material tested, with the lowest IC_50_ value toward osteoblast cells. We then demonstrated the possibility to associate such doped apatites with two biocompatible polymers, namely gelatin and poly(lactic-co-glycolic) acid PLGA, to prepare, respectively, composite 2D membranes and 3D scaffolds. The spatial distribution of the apatite particles and polymers was scrutinized by SEM and µCT analyses, and their relevance to the field of bone regeneration was underlined. Such bio-inspired antibacterial apatite compounds, whether pure or associated with (bio)polymers are thus promising candidates in dentistry and orthopedics while providing an alternative to antibiotherapy.

## 1. Introduction

Bone infections represent a major health challenge [1], especially taking into account the porosity of bone tissue that favors deep propagation of pathogens and compromise their eradication. In dentistry, for example, the high prevalence of periodontitis is considered as a major medically compromised condition in view of long-term efficient dental care and bone grafting, the success of which highly depends on whether the area of the surgery is infected or not [2]. Among frequent consequences of such infections are tooth loss and alveolar bone defects. Periodontal pathogens indeed induce a hyper-inflammatory response in the host that causes alveolar bone destruction [3]; and this is all the more problematic in tropical environments, where humid and warm conditions may promote fast bacterial development.

Present antimicrobial treatments often involve antibiotics that have progressively led to massive bacterial resistance effects with the emergence of multi-drug-resistant (MDR) microorganisms, counteracting antibiotic therapeutic efficiency [4]. It is indeed estimated that, by 2050, 10 million people could die in the world per year due to antibiotic resistance if no specific action is being undertaken [5]. There is therefore an urgent need for alternative antibacterial strategies. In clinical practice (such as in orthopedic, maxillofacial/dental, cranial, etc.), the possibility to use biomaterials with intrinsic antimicrobial properties is particularly appealing so as to treat the infection locally, right on the surgical site where pathogens can enter and disseminate [6,7]. This also may help reduce the administered dose of the active agent to the patient by more precisely delivering it to the site of interest rather than by the systemic route. Based on this context, the development of antibacterial biomaterials involving non-antibiotic active species becomes critical. This is particularly relevant in tropical environments where infections can rapidly propagate, e.g., in dentistry after tooth extraction and/or implant positioning. Furthermore, the direct contact with saliva—a body fluid often involving several bacterial strains—is an additional complexity.

In the field of bioactive materials, bone-biomimetic apatites are particularly appealing candidates as bioactive bone substitutes due to their composition and structure very close to those of natural bone mineral [8,9]. In contrast to stoichiometric well-crystallized hydroxyapatite (HA) that has long been used in orthopedics but which is not resorbable nor osteoinductive, biomimetic nanocrystalline apatites were shown to present exceptional bioactive properties [10,11,12,13,14], and their resorption rate can be modulated via their conditions of formation upon adjusting their maturation state [15,16]. Furthermore, biomimetic apatites can accommodate a large variety of ions and/or adsorb numerous types of drugs and (bio)molecules so as to convey “à la carte” additional properties such as anti-tumoral [11,13,14,17], anti-resorptive [18], or antimicrobial [19,20,21]. Association of antibiotics to calcium phosphate-based biomaterials has been the object of several works [22,23,24,25] including those with biomimetic apatites [12]. The local delivery may allow limiting the doses of antibiotics administered and make them more efficient on the surgical site. However, as mentioned above, due to bacterial resistance phenomena, it is essential to develop in parallel other strategies. The use of antibacterial metal ions (Ag^+^, Cu^2+^, Zn^2+^…) is one appealing alternative to antibiotics. Silver cations (Ag^+^) are for example well-known antibacterial species that proved their activity on several instances, including in associatioin with calcium phosphates [26,27,28,29,30], both on Gram negative and Gram positive bacteria. However, this ion is not naturally present in vivo and may lead to toxicity effects at certain doses. Anions have much less been investigated to this day in the context of antibacterial biomaterials; however, thanks to their potentiality to contain oxygen atoms and exhibit oxidizing properties, they could represent another appealing approach for providing antimicrobial properties to medical devices. Indeed, several natural defense mechanisms of the human organism are based on oxygenated chemical species in combination with the activity of specific enzymes such as peroxidase that may regulate their action under the control of the host body [31]. By providing local oxidizing conditions, these oxygenated species may neutralize pathogenic agents while being safe for the organism. Such oxidizing local conditions could be for example very relevant against anaerobic bacteria which may develop deep into teeth roots or bone tissue. Furthermore, oxygenation of tissues is essential for long-term functionality and to promote tissue healing [32]. An example of such oxidizing chemical species is the peroxide ion. It is found, for example, in the form of hydrogen peroxide as a household disinfectant or for water decontamination and is known for its antimicrobial properties [33,34], including in combination with other active agents such as Ag^+^. However, despite this apparent relevance, the use of peroxide ions as antibacterial agents in the composition of bone biomaterials still has to be explored.

In view of the above, the present work aims to develop safe-by-design antibacterial biomaterials based on a bio-inspired strategy, involving bone-like biomimetic apatites substituted with peroxide ions (O_2_^2−^) with the idea to evaluate, for the first time, their relevance as antimicrobial systems. To this aim, their effect against four relevant bacterial strains in bone infections and periodontitis was examined. In addition, co-doping with silver ions was evaluated for comparative purposes. Protein adhesion, osteoblast cell adhesion and viability were moreover evaluated to assess the biocompatibility of these bio-inspired antibacterial compounds. Finally, the possibility to process such bio-inspired doped apatites into actual 2D membranes or 3D scaffolds, eventually implying biodegradable polymers, was explored to widen the potential applicative spectrum of these novel compounds.

## 2. Materials and Methods

### 2.1. Synthesis of Apatite Powders

The synthesis protocol used to prepare peroxide-doped biomimetic apatites has been inspired from a preliminary physicochemical work [35]. Briefly, a calcium nitrate solution (75 mL, 0.3 M, Ca(NO_3_)_2_·4H_2_O, Merck, Darmstadt, Germany) was prepared in a mixture of hydrogen peroxide H_2_O_2_ (from a stock solution at 30%, VWR (Merck, Darmstadt, Germany) and water in variable final H_2_O_2_ proportions, i.e., 10, 25, and 50% respectively. This solution was mixed rapidly with a solution of di-ammonium hydrogenphosphate (150 mL, 0.6 M, (NH_4_)_2_HPO_4_, VWR (Merck, Darmstadt, Germany) prepared in the same H_2_O_2_/H_2_O proportions. The precipitate was then left to mature for 1, 3, or 7 days at room temperature, and then filtered through a Büchner funnel and freeze-dried. Silver and peroxide co-doped biomimetic apatites were prepared in the same conditions but replacing 2 mol.% of calcium nitrate by silver nitrate (AgNO_3_, Alfa Aesar, Erlenbachweg, Kandel, Germany). For comparative purposes, an Ag-only apatite sample (without peroxide) was also prepared, in pure water. Finally, an Ag-free and peroxide-free apatite control was synthesized as a control for the biological evaluations.

In the rest of the study, the following notations will be used: “hap” denoting the control undoped biomimetic apatite, “Ag-hap” denoting silver-only doping, “O_2_-hap” for peroxide-only doping, and “Ag-O_2_-hap” for co-doped silver/peroxide apatites.

### 2.2. Materials Fabrication

Compressed pellets: purely inorganic, compressed pellets were prepared out of the four types of apatite compounds (hap, Ag-hap, O_2_-hap and Ag-O_2_-hap) starting from the obtained freeze-dried powders. Pellets of each composition were prepared, with final dimensions of ca. 5 mm (diameter) × 2.5 mm (height). These compacted pellets were used especially for biological testing. Shaping from powders to pellets was processed by uniaxial pressing at RT using a Hounsfield press model H25K-S (Hounsfield, Redhill, UK) equipped with a calibrated 25 kN force sensor (France Scientifique, St Genis Laval, France). A 5-mm mold was used for all experiments, filled subsequently with 40 mg of each apatite power, and thoroughly washed with water and ethanol between each type of compound. The mechanical force (785 N or 40 MPa) was applied vertically at a controlled speed of 10 mm/min to allow optimized particles re-arrangement, and a dwell time of 20 s was maintained to obtain final compacted 3D pellets with intrinsic cohesion allowing easy manipulation, including in culture media.

Two-dimensional and three-dimensional composites associating the apatites and biocompatible polymers were produced on the basis of a freeze-casting methodology:

Two-dimensional membranes: the 2D membranes were prepared by associating the apatite powders with gelatin. 37 mg of trisodium citrate (Merck, Darmstadt, Germany) were dissolved homogenously in 10 mL distilled water. Then 0.6 g type-B gelatin (Nitta Gelatin, Osaka, Japan) was added to the solution and the gelatin was let to swell for 30 min. The solution with the added swelled gelatin was heated at 37 °C and stirred at 200 rpm for 30 min. An amount of 0.4 g apatite sample was added while stirring was continued at 300 rpm for 2 h. During the process, pH was adjusted at 7.0. After 2 h of maturation, 1 g of solution was poured into a balance dish (4 cm × 4 cm). The balance dish with the poured solution inside was frozen at −20 °C for 24 h, followed by 18–24 h lyophilization. The obtained membranes were then crosslinked dehydrothermally in a vacuum oven at 140 °C for 72 h [36], and punched into 6 mm diameter pieces. Such punched membranes were packed in a medical pouch and sterilized with ethylene oxide gas at 36.5 °C for 7 h until further use. This procedure was repeated for all 4 types of apatite compounds studied in this work.

Three-dimensional scaffolds: the 3D porous scaffolds were produced by freeze-casting using a homemade system, comprising a cylindrical mold (internal diameter 2 cm, height 5 cm) closed by top and bottom copper stoppers. In particular, the bottom copper socket allowed for efficient heat transfer. The mold was isolated from the environment via an insulating polymeric foam jacket. Control of the freezing front inside the mold was made possible by setting the temperature of the bottom socket connected to a refrigerating device. The temperature was set to −10 °C and verified by way of two thermocouples. The composite scaffolds were prepared by association of the O_2_-hap apatite sample with poly (D,L-lactic-co-glycolic acid), PLGA (Merck, Darmstadt, D-lactic/L-lactic/glycolic acid: 25/25/50 with Mn = 23,000 g.mol^−1^ and Ð = 1.52). The latter was dissolved in dimethyl carbonate (DMC) to prepare a solution at 3.5 wt.%. The mass ratio between apatite (in powder form) and PLGA was set to 40:60. After the addition of the apatite particles into the solubilized PLGA, the suspension was homogenized by vortex and sonication (2 min). The suspension was then poured into the cylindrical mold and left to freeze-cast for 5 h. Molds were then opened and the frozen suspension was lyophilized for 24 h (Christ Alpha 2-4 LD2, Osterode am Harz, Germany) at −75 °C, 0.05 mbar.

### 2.3. Physicochemical Characterization

The apatite structure of the samples was verified by X-ray diffraction XRD using an INEL Equinox 1000 diffractometer (INEL, Artenay, France) with a cobalt anticathode (λ_Co_ = 1.78892 Å). Complementary analyses were carried out by Fourier transform infrared spectroscopy FTIR with a Nicolet IS50 spectrometer (Thermo Scientific, Waltham, MA, USA) in the 400–4000 cm^−1^ wavenumber range and a resolution of 4 cm^−1^.

The elemental composition of the apatite was determined by way of several analyses. Calcium was titrated by atomic absorption spectroscopy AAS using a ContAA 300 spectrophotometer (Analytik Jena, Jena, Germany). Analyses using a N_2_O/C_2_H_2_ flame were made at pH ~2 in the presence of HNO_3_ (0.06 M, VWR, Merck, Darmstadt, Germany) and CsCl (SCP Science, Québec, Canada) at 2 g/L and La(NO_3_)_3_ (SCP Science, Québec, Canada) at 6 g/L for analysis optimization. Calibration (R^2^ = 0.9964) and measurements were run in the range 0–3 ppm. Similarly, silver was titrated by AAS using an air/C_2_H_2_ flame, in the range 0–7.5 ppm but in the absence of CsCl to avoid silver chloride precipitation (calibration R^2^ = 0.9941). Phosphorus contents were measured in the range 0–25 ppm by visible spectrophotometry (Shimadzu 1800, Kyoto, Japan) by following, at λ = 460 nm, the absorbance of the phospho-vanado-molybdenic complex formed upon addition of an excess of ammonium vanadate and ammonium molybdate (calibration R^2^ = 1.0000). Peroxide titration was carried out by manganimetry using the following equation:

(1) $$2MnO_{4}^{-}+16H^{+}+5O_{2}^{2-}\to 2Mn^{2+}+8H_{2}O+5O_{2\left(g\right)}$$

where the equivalence is reached, in acid conditions (H_2_SO_4_, VWR, Merck, Darmstadt, Germany), upon stable purple coloration of the solution due to an excess of permanganate ions. The stock solution of KMnO_4_ (AVS, New Jersey, USA) was checked by titrating a freshly opened stock solution of H_2_O_2_ (30%, VWR, Merck, Darmstadt, Germany).

Scanning electron microscopy SEM on raw apatite samples was performed using an FEI Quanta 450 microscope (FEI, Oregon, USA) operated at 12 kV voltage and in low vacuum mode. SEM analyses were carried out on the bottom surface of membranes and on the median zone of transversal cross-sections for scaffolds.

X-ray computed microtomography µCT images were acquired to evaluate the three-dimensional morphology and multiscale organization of the 2D membranes and 3D scaffolds. A Phoenix/GE Nanotom 180 instrument (GE Sensing & Inspection Technologies GmbH, Phoenix|x-ray, Wunstorf, Germany) using a tungsten target (mode 0) was used at a voltage of 50 kV and a current of 300 µA. Samples were positioned respectively at 25 mm (membranes) and 40 mm (scaffolds) from the X-ray target. A distance of 300 mm was used between the source and the detector, with a counting time of 750 ms per picture and an average of five pictures per 0.25° step. The Datos X^®^ software (Version 1.5, GE Sensing & Inspection Technologies GmbH, Phoenix|X-ray, Wunstorf, Germany) was used to process the data and reconstruct 3D images of the scaffolds. Images were treated using the Vg Studio Max^®^ software (Version 2.1 max, Volume Graphics GmbH, Heidelberg, Germany). The maximum voxel size was respectively 6 μm (membranes) and 4.2 μm (scaffolds).

### 2.4. Antibacterial Properties

Antibacterial tests were carried out using the following equipment: a pipette to collect bacteria, a test tube for liquid brain heart infusion (BHI), a sterile loop to apply bacteria, disks, a refrigerated centrifuge (Eppendorf 5417 R, Hamburg, Germany), laminar flow (Labonco Co, Kansas City, MO, USA), an incubator (Sanyo MIR 162, Osaka, Japan), an autoclave for Tryptone Soya Agar (TSA) sterilization, a digital caliper to measure the TSA radical zone, and a lamp.

Four relevant types of bacteria were considered in this work: *Porphyromonas gingivalis* (ATCC 33277), *Aggregatibacter actinomycetemcomitans* (ATCC 43718), *Fusobacterium nucleatum* (ATCC 25586), and *Staphylococcus aureus* (ATCC 6538). The first 3 types of bacteria are black pigmented anaerobic Gram negative bacteria that are the main cause in the development of periodontitis [37]. *S. aureus* is a highly pathogenic Gram positive bacteria involved in many bone-related infections and studies.

The antibacterial activity of the apatite samples was carried out in agar plates. In a first stage, the tryptic soy agar (TSA) media were prepared by weighing TSA stock (heated to dissolve in distilled water) and distributing it to the tubes to prepare agar media with the same thickness of approximately 6 mm. The TSA media in the tubes were then sterilized in an autoclave at 121 °C temperature and 1 atm pressure for 15 min. The tubes were then poured into sterile petri dishes with the same diameter and the plates were allowed to cool and solidify. After completing the preparation of TSA media and bacteria suspension, the TSA media were perforated to create wells (5 mm depth with 5 mm diameter).

Meanwhile, a suspension of *P. gingivalis*, *A. actinomycetemcomitans*, *F. nucleatum*, and *S. aureus* was made, and then the cultured colonies were contacted with 0.5 mL of liquid BHI, and then incubated for 5–8 h at 37 °C. Sterile distilled water was added to a certain turbidity in accordance with the standard germ concentration of 108 CFU per mL (CFU, Colony Forming Unit). Simultaneously, 4 specimen pellets (hap, Ag-hap, O_2_-hap, and Ag-O_2_-hap) were prepared in 5 mm diameter with 1 mm thickness and sterilized with dry low temperature ethylene oxide gas (EOG).

**Figure 1:**
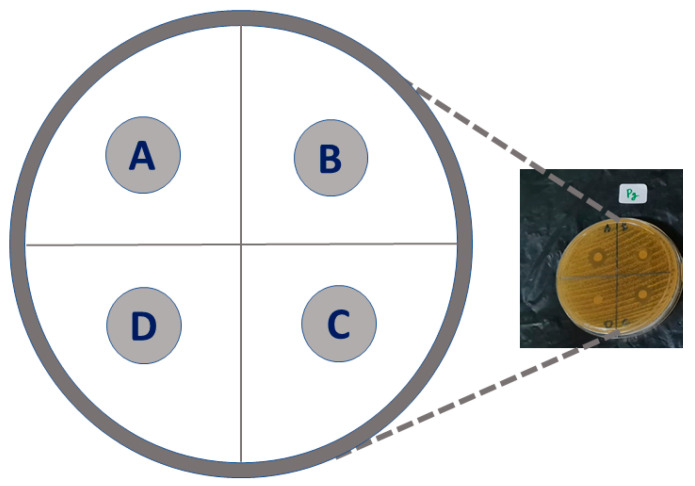
Schematic diagram for antibacterial tests arrangement. The 4 types of bacteria were incubated in a Petri dish, with Ag-hap (A), O_2_-hap (B), Ag-O_2_-hap (C), and hap as the reference (D).

A sterile cotton swab was dipped in the germ suspension, pressed against the tube wall until the cotton was wet, then smeared on the surface of each media evenly. The sterile hap, Ag-hap, O_2_-hap and Ag-O_2_-hap pellets were introduced in the wells as depicted in Figure 1. The media with bacteria and 4 types of apatite specimens were incubated at 37 °C for 24 h. Each sample was tested in quadruplicate (*n* = 4). The test results were analyzed by measuring radical zones, e.g., the clear areas formed around the well with a sliding caliper.

Data of the bacteria inhibition zone in mm are presented as the mean ± standard deviation (SD). Statistical analysis was conducted with a one-way analysis of variance (ANOVA) continued by Bonferroni post-hoc test.

### 2.5. Protein Adsorption

Protein adsorption onto the apatite scaffolds was determined using a method adapted from the literature [38,39]. Briefly, powders (hap, Ag-hap, O_2_-hap and Ag-O_2_-hap) were weighed with masses of 0.1 g for each sample. Before incubation, the samples were immersed in 1 mL of 70% ethanol (Merck, Darmstadt, Germany) for 1 h and then washed three times with PBS. The samples were incubated in 1 mL PBS containing 10% FBS for 1 h at 36.5 °C. After incubation, the PBS containing 10% FBS were taken out from the tube and put into a 15 mL conical tube. The samples were then immersed in 1 mL PBS for 15 min to remove the proteins that were not completely attached to the samples surface. The PBS which was used to remove proteins that were not completely attached to the samples surface was added to the conical tube containing 1 mL PBS containing 10% FBS. The immersion in 1 mL PBS for 15 min was repeated three times, and all the washing solutions were added to the remaining PBS containing 10% FBS solution from the incubation. Thus, there were around 4 mL solutions in the 15 mL conical tube which were ready for the analysis. The protein remaining in the solution was quantified by UV spectrophotometry (Shimadzu 1800, Kyoto, Japan) at λ = 280 nm. The amount of protein adsorbed on the surface of the samples was calculated via a differential approach by subtracting the protein concentration after incubation from the protein concentration before incubation.

### 2.6. Osteoblast Cell Viability

Cell viability was studied by 3-(4,5-dimethylthiazol-2-yl)-2,5-diphenyltetrazolium bromide (MTT) assay. A series of samples (hap, Ag-hap, O_2_-hap, and Ag-O_2_-hap) in solution with different concentrations of 31.25, 62.50, 125, 250, 500, 1000, 2000, and 4000 mg/mL were prepared by suspending the samples into distilled water at 60 °C temperature at 350 rpm velocity for 1 h until it turned into a homogenous suspension, which was sonicated before storage in the refrigerator.

Mouse osteoblast cells (osteoblast-like cells MC3T3E1) were cultured in MEM-α medium (Gibco, CA, USA) with additional 10% FBS (Gibco, CA, USA) enriched with 2% Penicillin-Streptomycin (Gibco, CA, USA) and 0.5% Fungizone (Gibco, CA, USA). The cell suspension in 100 µL were seeded on the bottom of a 96-well plate at a density of 2 × 10^4^ cells/well. After that, the cells were incubated for 24 h at 37 °C in 5% CO_2_. An amount of each 100 mL of the sample solution was added into the wells, with control (the well with cells without sample solutions). Again, the incubation was conducted for the next 24 h at 37 °C in 5% CO_2_ incubator.

Cell viability was studied by MTT assay for each period of incubation (24 h). The measurement was conducted in triplicate for each type of sample (hap, Ag-hap, O_2_-hap, and Ag-O_2_-hap) and a control (the well without sample solution). For MTT assay, the medium was discarded, 100 μL of MTT (Biobasic, ONT, Canada) solution with a concentration of 0.5 mg/mL was added to the well and incubated for 4 h. Then, dimethyl sulfoxide DMSO (Merck KGaA, Darmstadt, Germany) was added to the well at 100 μL/well. The absorbance was recorded by Tecan Spark^®^ (Tecan Trading AG, Zurich, Switzerland) at λ = 570 nm [39,40]. The cell viability was calculated by the following equation:

(2) $$\mathrm{Cell}\;\mathrm{viability}\;\left(\%\right)=\frac{\left(\mathrm{Absorbance}\;\mathrm{of}\;\mathrm{the}\;\mathrm{scaffold}\;-\;\mathrm{Absorbance}\;\mathrm{of}\;\mathrm{medium}\;\mathrm{control}\right)}{\left(\mathrm{Absorbance}\;\mathrm{of}\;\mathrm{the}\;\mathrm{control}-\mathrm{Absorbance}\;\mathrm{of}\;\mathrm{medium}\;\mathrm{control}\right)}\times 100$$

All tests were conducted at least in triplicate. The mean values of cell viability with the 4 types of apatites were calculated using Microsoft Excel (Version 365, Microsoft, Washington, USA). The IC_50_ value was analyzed by linear regression. Data are presented as means ± standard deviation (SD). A one-way analysis of variance (ANOVA) with the GraphPad Prism 7 software (GraphPad Software, Version 9.3.1, Graphpad Software Inc., CA, USA) was used to analyze the results followed by Tukey’s test at *p*-values < 0.05.

### 2.7. Osteoblast Cell Adhesion

Using the same procedure as for the MTT assay, MC3T3E1 mouse osteoblast cell adhesion tests were conducted. In this case, the assay was applied with CCK (Cell Counting Kit) containing 2-2methoxy 4 nitrophenyl 3-4 nitrophenyl 5,2,4 disulophenyl 2H tetrazolium monosodium salt (Merck, Germany). The CCK reagent was used to allow further incubation of the cells with the samples for different periods. Briefly, the CCK reagent was prepared in a complete medium to transfer 100 µL of it into each well and incubate for 1 h. The absorbance was recorded by Tecan Spark^®^ (Tecan Trading AG, Switzerland) at λ = 450 nm. The mean values of the cell attached to the samples were calculated with Equation (2) and one-way ANOVA with the GraphPad Prism 7 software (GraphPad Software, Version 9.3.1, Graphpad Software Inc., CA, USA) was also applied to analyze the results. All tests were conducted at least in triplicate. Data are presented as means ± standard deviation (SD). A one-way analysis of variance (ANOVA) was used to analyze the results followed by Tukey’s test at *p*-values < 0.05.

The spreading of the osteoblast cells on the surface of the apatite materials was visually observed by SEM analysis. To this aim, MC3T3E1 cells were seeded on the doped apatite materials and incubated for 48 h. After 48 h of incubation, the culture was washed with PBS 3 times and fixated with 2.5% glutaraldehyde. Dehydration series with alcohol were conducted at 50, 70, and 90% concentration before final dehydration with 100% alcohol, 2 times for 30 min each. Specimens were then dried at room temperature, platinum coated, and observed with a JEOL-JSM 6510 microscope (JEOL Ltd., Tokyo, Japan).

## 3. Results and Discussion

### 3.1. Preparation and Characterization of Doped Biomimetic Apatites

In a previous study, some of us (Drouet’s group) explored strategies to prepare peroxide-doped biomimetic apatites either from hydrolysis of β-TCP in the presence of hydrogen peroxide H_2_O_2_ or by modifying a protocol of preparation of biomimetic apatites by adding H_2_O_2_ to the medium [35]. Only this second route allowed us to obtain single-phased nanocrystalline apatites that exhibited a biomimetic character with a wide range of H_2_O_2_ proportions in the precipitating medium. These findings served as basis for the present study aiming to explore their interaction with bone cells and their antibacterial potentialities. Here, we selected three starting H_2_O_2_ proportions in the medium, namely 10, 25, and 50%, and three maturation times for the apatites, i.e., 1, 3, and 7 days. This led to nine peroxide-doped apatite compounds, as listed in Table 1.

In parallel, Ag-doped biomimetic apatites were also explored previously [41], unveiling that Ag^+^ ions conferred antibacterial activity, even at a low dose, on Gram negative (*E. coli*, *P. aeruginosa*) and Gram positive (*S. aureus* and *S. epidermidis*) bacterial strains. On the basis of this background work, we chose here to incorporate ~0.15 moles of Ag^+^ per unit formula in the silver-doped apatites, representing about 2 mol.% of replacement of Ca^2+^ ions. Thus, nine silver-peroxide co-doped samples were additionally prepared (Table 1). Finally, a peroxide-free sample containing only silver (Ag-hap) as well as an undoped apatite sample (hap) were prepared for comparative purposes.

**Figure 2:**
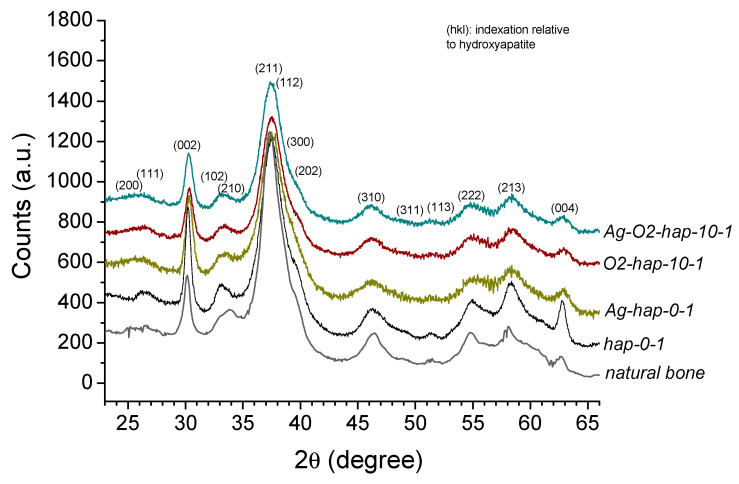
XRD patterns for selected peroxide and/or silver-doped and undoped apatites, compared to natural bone (rat, nine months old, CIRIMAT internal specimen). The main (khl) diffraction lines corresponding to the hydroxyapatite phase (PDF file #00-009-0432) are also annotated.

The apatitic nature of the samples was verified by XRD analyses. Figure 2 illustrates as typical examples the XRD patterns obtained for the four selected samples envisioned for in vitro testing. Additional XRD patterns of samples prepared with varying H_2_O_2_ initial contents (10, 25, or 50 vol.%) and maturation times (1, 3, or 7 days) are presented as Supporting Information in Figure S1. As may be seen, all samples displayed the characteristic apatite signature, as evidenced by comparison to the (khl) main diffraction lines of hydroxyapatite (PDF file #00-009-0432). The increase in H_2_O_2_ initial content in the precipitating medium (Figure S1a, for 1 day of maturation) had only a very limited effect on the recorded patterns, pointing to rather similar crystallinity states. An increase in apatite maturation time from 1 to 7 days for a fixed H_2_O_2_ initial proportion (Figure S1b, for 50 vol.% H_2_O_2_) only led to a slight increase in crystallinity, as indicated from the better resolution of the XRD pattern, e.g., around line (202). It may be remarked from Figure 2 that a good correspondence may be observed between all selected samples and natural rat bone (nine months old, internal specimen of the CIRIMAT laboratory, University of Toulouse, France), thus evidencing their mimetic character to bone apatite.

Complementary analyses were run by FTIR. Again, the features obtained for the samples were highly similar. Figure S2 shows the example of apatites prepared with increasing H_2_O_2_ initial amounts for a maturation time of 1 day. The presence of HPO_4_^2−^ ions was in particular noticed, typically around 875 (noted νHPO4 in Figure S2) and 535 cm^−1^ (noted ν’ HPO_4_), thus evidencing nonstoichiometry for bone apatite [8,42].

The chemical contents of the prepared apatites were measured experimentally in terms of calcium, phosphate, silver (Ag^+^), and peroxide (O_2_^2−^) ions, by way of complementary techniques (AAS for Ca and Ag, visible spectrophotometry for P, and manganimetry for peroxide). Ag^+^ and O_2_^2−^ contents are in particular relevant in this study on antibacterial materials, and have been added in Table 1. For all Ag-loaded compounds, a rather stable content of ~0.15 mole per unit formula was obtained throughout the samples, as expected, and their mean (Ca + Ag)/P mole ratios was of 1.40. In parallel, an increase in peroxide content, ranging from 0.39 to 0.66, was noticed upon increasing the starting H_2_O_2_ proportion in the precipitating medium and/or the maturation time of the apatite phase from 1 to 7 days. For Ag-free apatites, the mean Ca/P ratio was of 1.49. Since 1 day of maturation combined with 10 vol.% of initial H_2_O_2_ in the starting medium led to significant peroxide doping, these conditions were selected for the biological tests presented in the next sections (marked by an “X” symbol in Table 1). Indeed, in view of potential industrialization of the synthesis process, minimization of processing time and energy as well as of the amount of H_2_O_2_ reactant appeared to us as particularly relevant.

**Figure 3:**
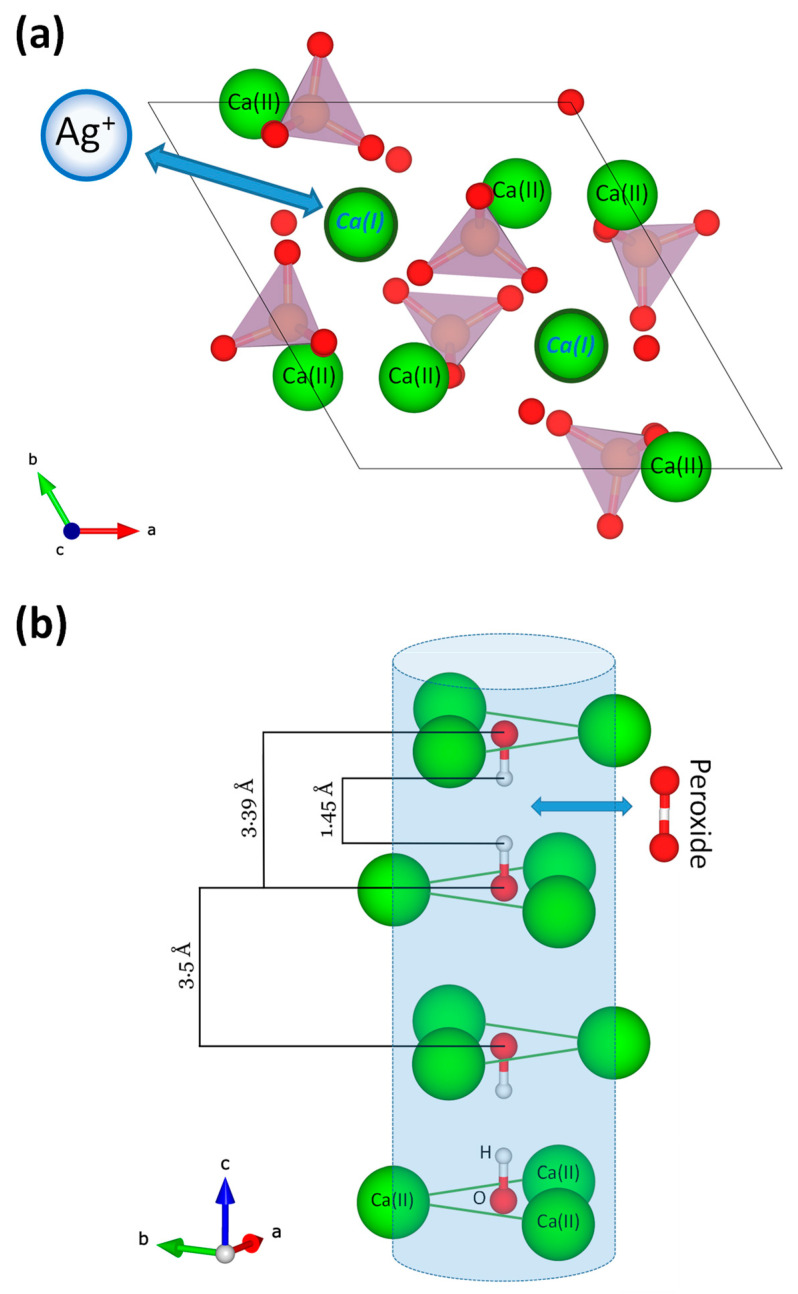
Structural arrangement of ions in the apatite structure. (**a**) Representation in the ab-plane showing cationic sites occupied by Ca^2+^ ions as Ca(I) and Ca(II) and the reported substitution of Ag^+^ in Ca(I) sites; (**b**) apatitic channels parallel to the c-axis and formed by Ca(II) sites. Some specific distances involving the O and H atoms in OH^-^ ions are indicated. The peroxide ion O_2_^2−^ is modeled considering the O–O bond length in H_2_O_2_ (close to 1.48 Å): 1 peroxide ion is hypothesized to replace two adjacent OH^−^ ions.

In such doped apatites, Ag^+^ cations are expected to occupy some of the calcium sites of the apatite network. Evidence of such Ag-for-Ca substitutions have been reported on several occasions [43,44,45,46]. Badrour et al. have proposed a positioning in place of Ca(I) crystallographic sites that we illustrate here in Figure 3a [45], and Lim et al. suggested that the electroneutrality was ensured by OH^-^ vacancies [46]. The possibility to have part of the silver localized as a separate phase, e.g., silver phosphate, on/between the apatite particles cannot be totally ruled out taking into account the affinity of these two ions; however, we did not find any evidence of the presence of such secondary phases in our XRD or FTIR analyses. In the case of peroxide ions, fewer data are available for substitutions in apatite. For peroxyapatites obtained by heat treatment of hydroxyapatite in oxygenated atmosphere, Zhao et al. have concluded on the substitution of OH^-^ ions from the apatitic channels formed by Ca(II) sites and parallel to the c-axis [47]. Although the mode of synthesis is different from the biomimetic approach used in the present work, we may hypothesize that similar partial substitution of OH^-^ ions by peroxide ions (1 O_2_^2−^ for 2 OH^−^) also explains the peroxidation effect observed here. Figure 3b represents the corresponding ion arrangement of the apatitic channels along with a peroxide ion to describe this substitution mechanism.

### 3.2. In Vitro Assessments

**Figure 4:**
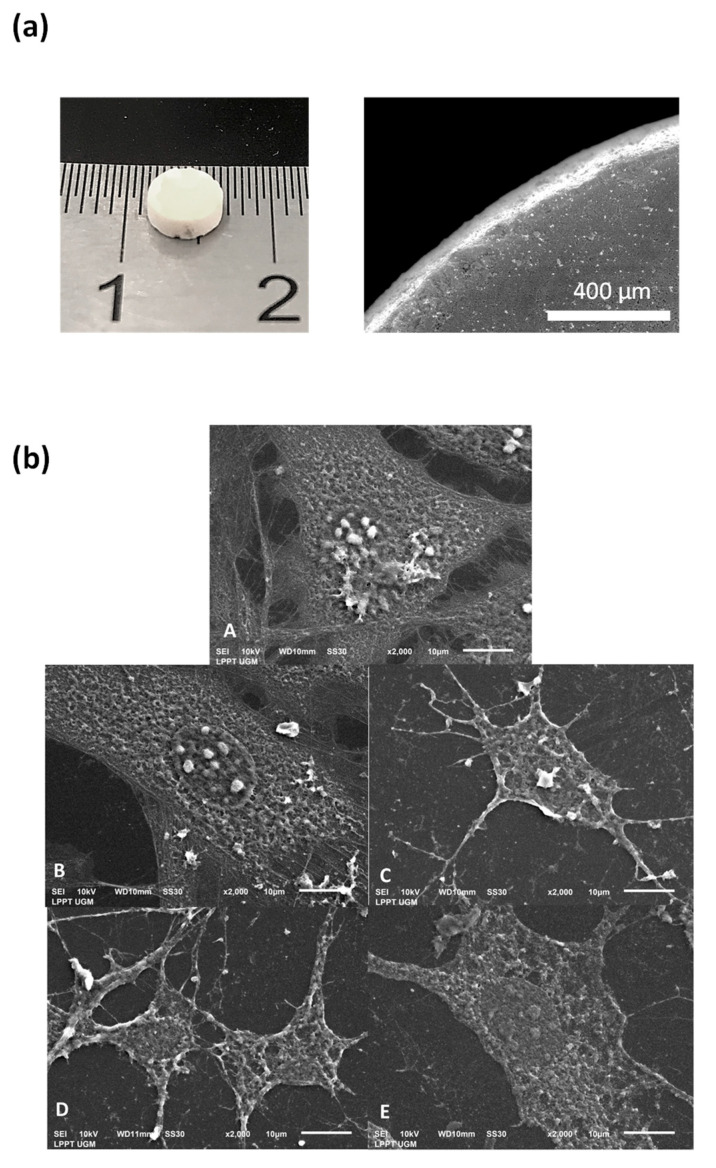
Observation of compacted apatite pellets: (**a**) Typical example of produced pellet: optical microscopy (left) and SEM (right) for the Ag-O_2_-hap sample); (**b**) adhered MC3T3E1 osteoblast cells on apatite pellets, where A is cell control, B is Ag-hap, C is O_2_-hap, D is Ag-O_2_-hap, and E is hap as reference.

In view of some of the biological tests planned in this study, compacted apatite samples were prepared by uni-axial compression of the starting powders at room temperature and under 40 MPa. Three-dimensional pellets of 5 mm diameter and 2.5 mm height were obtained (Figure 4a), exhibiting intrinsic cohesion to allow easy handling and subsequent in vitro cell tests in aqueous culture media. Twenty pellets of each condition (hap, Ag-hap, O_2_-hap, and Ag-O_2_-hap) were prepared, with a mean measured apparent density of 0.79 ± 0.05 g/cm^3^ among all samples.

The safe-by-design approach developed in this work lies in the association between biomimetic bone-like apatites and oxygenated doping ions (peroxides, in this study), the latter being antibacterial agents inspired from natural processes in vivo for fighting against pathogenic agents. Since the literature also unveiled possible synergistic effects with silver ions, Ag^+^ doping was also investigated. The following step therefore consisted of assessing whether such O_2_- and/or Ag-doped nanocrystalline apatites exhibited effective antibacterial properties.

Taking into account the clinical needs targeted in this work, the selection of bacteria for in vitro testing was made on the basis of a literature survey dedicated to periodontitis. Around 400 species of bacteria are found in periodontal pockets, but not all species are found in every individual. Eight bacterial species are consistently found in periodontitis, including *Porphyromonas gingivalis, Actinobacillus actinomycetemcomitans, Fusobacterium nucleatum, Tannerella forsythia, Treponema denticola, Eubacterium stainum, Prevotella intermedia*, and *Prevotella nigrescens* [48,49]. On the basis of this overview, we selected the three first bacterial strains cited above, namely *P. gingivalis*, *A. actinomycetemcomitans*, and *F. nucleatum*, as well as *S. aureus*. The first three types of bacteria are anaerobic, Gram negative, and often involved in the development of periodontitis; in particular, *P. gingivalis* is the main bacterium found in patients with periodontitis [37]. On the other hand, *S. aureus* is a major pathogenic Gram positive bacterium relevant to several bone infections and also appeared relevant in this work to investigate the potential of such peroxidated apatites.

**Figure 5:**
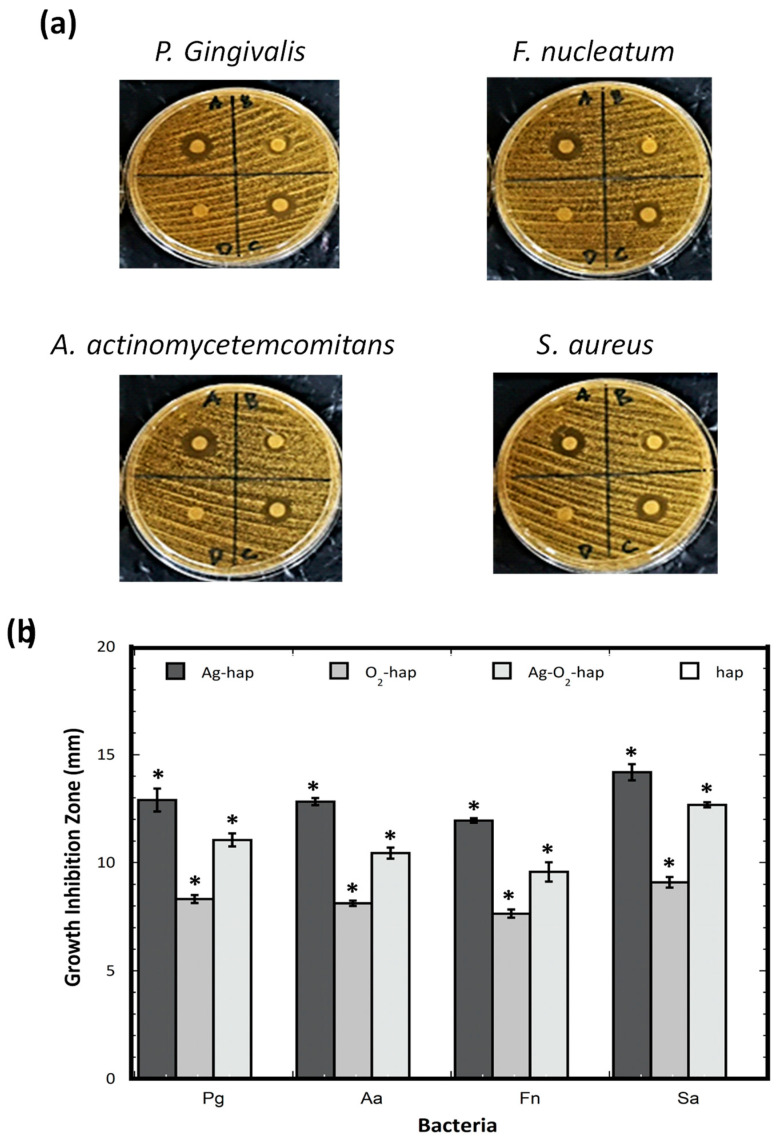
(**a**) Results of antibacterial tests with *P. gingivalis* (Pg), *A. actinomycetemcomitans* (Aa), *F. nucleatum* (Fn), and *S. aureus* (Sa) for (**b**) all four types of apatite samples: (A) Ag-hap, (B) O_2_-doped, (C) Ag-O_2_-hap, and (D) hap. The asterisk * shows statistically significant differences. The statistical ANOVA analysis is given in Tables S1 to S5 as supporting information .

Figure 5 reports the results obtained on the four types of apatites and the four types of bacteria. Interestingly, Figure 5a shows the existence of inhibition halos for all doped samples, demonstrating the antibacterial role of the peroxide and the silver ions, taken separately or combined. In contrast, undoped hap did not exhibit any detectable effect, thus confirming that the growth inhibition could be assigned to the presence of the doping ions. Figure S3 reports the detailed quadruplicate replication of these conditions, showing good reproducibility, and the corresponding quantitative data analysis (one-way ANOVA) is given for each type of bacteria in Tables S2 to S5. Post-hoc tests also confirmed that there were significant differences among the samples on the capability of the samples to function as antimicrobial agents.

Evidence of antibacterial properties assignable to peroxide ions in peroxidated apatites confirms the relevance of the safe-by-design approach pursued in this work, as demonstrated here not only in relation with the three anaerobic bacteria tested, but also for S. aureus, a major pathogen in bone infections. Compared to O_2_-hap, the co-doped Ag-O_2_-hap samples showed an even greater antibacterial power, thus evidencing the additional well-known antibacterial properties of Ag^+^ cations. It may be noted that this effect was, however, lower than for the Ag-only doped sample (Ag-hap); therefore, in this work, we did not observe synergistic antibacterial effects of O_2_^2−^ and Ag^+^ ions.

Besides antibacterial properties, it is essential to investigate also the behavior of eukaryotic cells and demonstrate the absence of significant toxicity. In this work, osteoblast cells were selected with this aim, taking into account the bone-related applications. Generally speaking, cell behavior is highly related to the surface features of the biomaterial and to the adsorption of proteins which represents an important step following implantation. Protein adsorption on a biomaterial may indeed contribute to control or orient cell-material interaction and in turn biological processes involving biochemical signaling, biodegradation, etc. Protein adsorption evaluation is thus a relevant complement to biocompatibility and bioactivity assessments in the development of biomaterials. Cell activity on bioceramic surfaces was for instance shown to be influenced by fibronectin adsorption, a key plasma protein, in terms of osteoblasts and progenitor cell adhesion [50,51].

**Figure 6:**
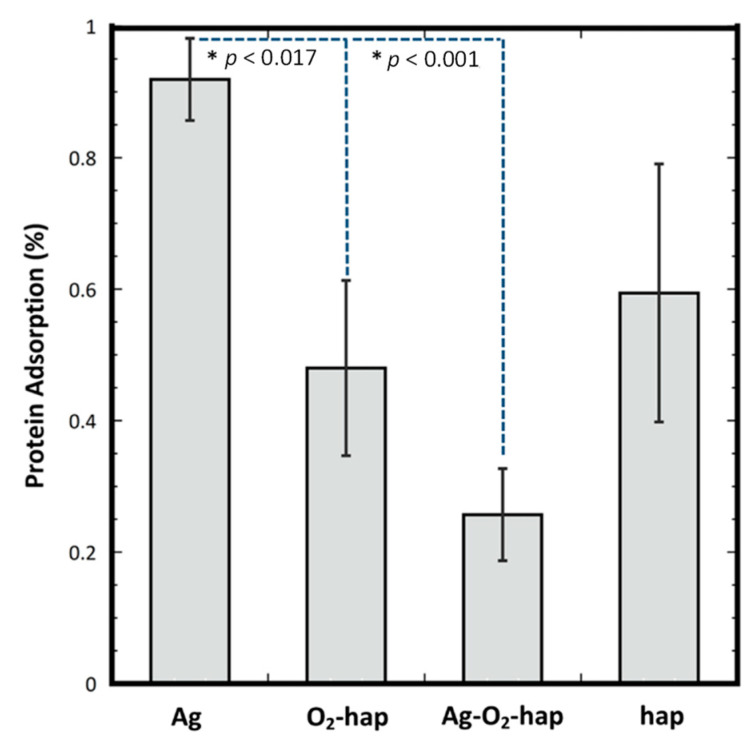
Protein adsorption quantification (after contact with Fetal Bovine Serum) on the four types of apatite samples. The statistical ANOVA analysis is given in Table S6 as supporting information.

In the present work, the level of protein adsorption reached for each type of apatite samples after contact with Fetal Bovine Serum (FBS) was quantified via a differential approach by UV spectrophotometry (see experimental data for details). Figure 6 shows the results obtained, and the numerical values from the ANOVA analysis are reported in Table S6. Peroxidated apatite O_2_-hap showed a similar adsorption level compared to undoped apatite, as no significant difference was found between Ag-hap and hap (*p* = 0.084), thus not modifying significantly surface interaction with circulating proteins. Samples involving silver showed however different behaviors. Indeed, there were significant differences on the protein adsorption between Ag-doped with Peroxide-doped (*p* < 0.017) and between Ag-doped with Ag-Peroxide-doped (*p* < 0.001). This led to the following order: Ag-hap > O_2_-hap ≅ hap > Ag-O_2_-hap. The presence of Ag^+^ ions thus seems to affect proteins’ interaction with the sample surfaces. Ag-for-Ca substitution could generate modifications of the surface features such as the surface charge, although in a way that varies depending on the copresence of peroxide ions or not. Although signs of secondary phases were not seen by XRD nor FTIR for the co-doped Ag-O_2_-hap sample, the simultaneous presence of Ag^+^ and O_2_^2−^ (oxidizing) ions may have led to the combination of these ions to some extent on the surface of the apatite particles, generating silver (per)oxide, thus modifying the surface features; indeed, such phases as silver peroxide may exhibit different surface charges and ionic mobility compared to regular apatite. Furthermore, this hypothesis might explain the peculiar antibacterial behavior observed for the co-doped sample Ag-O_2_-hap in Figure 5 (on the basis of modified availability of Ag^+^ ions after immersion). We may however underline that SEM-EDX analyses performed (Figure S4) on the four types of apatite powders did not show any signs of Ag clustering (even in BSE mode), thus the possible difference of Ag^+^ ions environment does not appear to be explainable by abnormal silver concentration at the surface of the particles.

**Figure 7:**
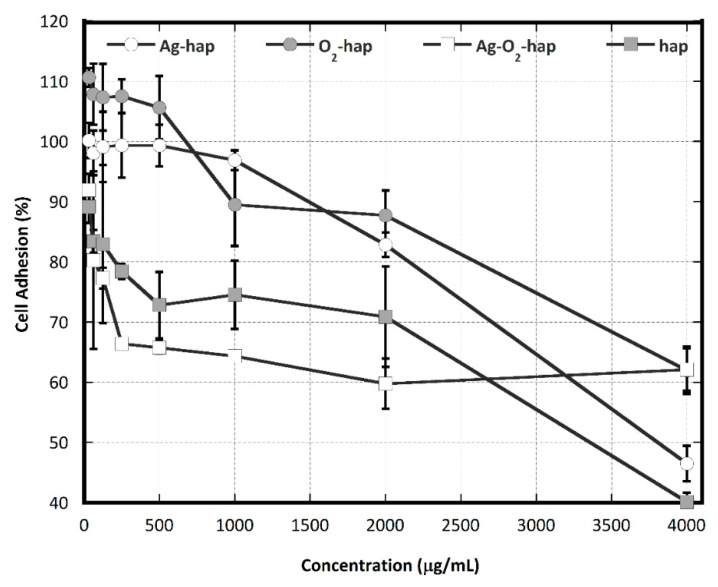
MC3T3E1 osteoblast cell adhesion data (after samples incubation for 24 h) versus the concentration of the four types of apatite samples, taken separately. The statistical ANOVA analysis is given in Table S7 as supporting information.

In the next step of the study, the adhesion of osteoblast cells (MC3T3E1, mouse lineage) was explored (Figure 7 and quantitative ANOVA analysis in Table S7). From the statistical ANOVA analysis, O2-hap and Ag-hap exhibited the highest degree of cell adhesion, without significant difference (*p* = 0.635). For both of these samples, the percentage of adhered cells was still >80% even at the concentration of 2000 µg/mL. It may be noted that for concentrations <1000 µg/mL, the O2-hap sample also allowed osteoblast cells to start proliferating, as shown by increased numbers beyond 100%, thus demonstrating a welcoming surface for osteoblasts. Statistical differences were found between Ag-hap with Ag-O_2_-hap (*p* < 0.01), Ag-hap with hap (*p* < 0.01), O_2_-hap with Ag-O_2_-hap (*p* < 0.01), and O_2_-hap with hap (*p* < 0.01). Despite the peculiar behavior of the co-doped Ag-O_2_-hap sample at very high concentrations (4000 µg/mL), cell adhesion followed the general trend: O_2_-hap ≅ Ag-hap > hap > Ag-O_2_-hap. Apart from the “improved” behavior of O_2_-hap, this order resembled the one obtained for protein adhesion which is often a parameter related to cell attachment. Of course, other parameters are likely to come into play as well, such as the surface topography and the potentiality to release ions upon immersion, among others.

**Figure 8:**
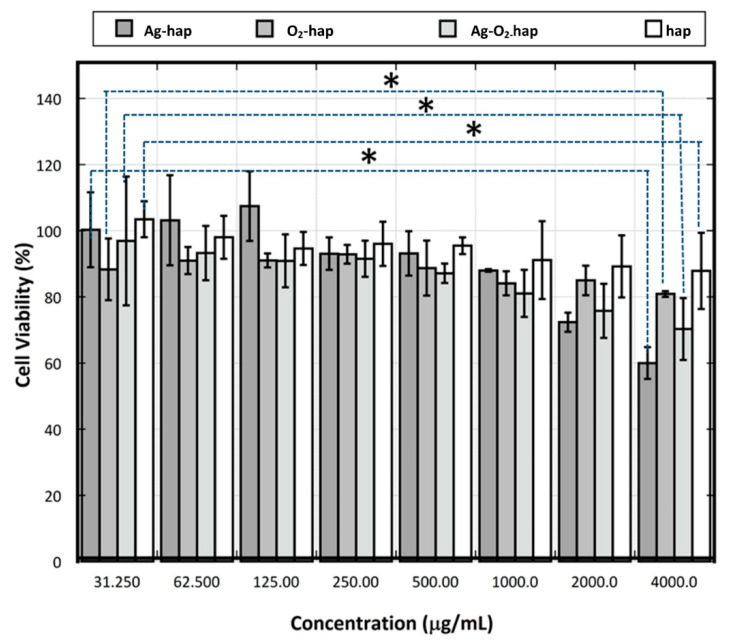
Osteoblast cell viability. The asterisk * shows statistically significant differences. The horizontal line shows the 70% viability limit.

Cytotoxicity testing is designed to determine whether a biomaterial may cause acute toxicity. For biocompatibility evaluations, mammalian cell culture is the most frequently used approach in vitro [52]. In this study, we checked cytotoxicity by counting the percentage of viable osteoblast cells. Results (Figure 8) showed that all apatite samples tested, whether doped or not, allowed high cell viability. Indeed, viability levels above 70% were found in all cases—except for in elevated concentrations like 4000 µg/mL for Ag-containing samples. No significant differences of cytotoxicity could be assessed among the different types of samples, only a slight decrease was noted upon concentration increase, as is customary in biomaterial testing.

**Figure 9:**
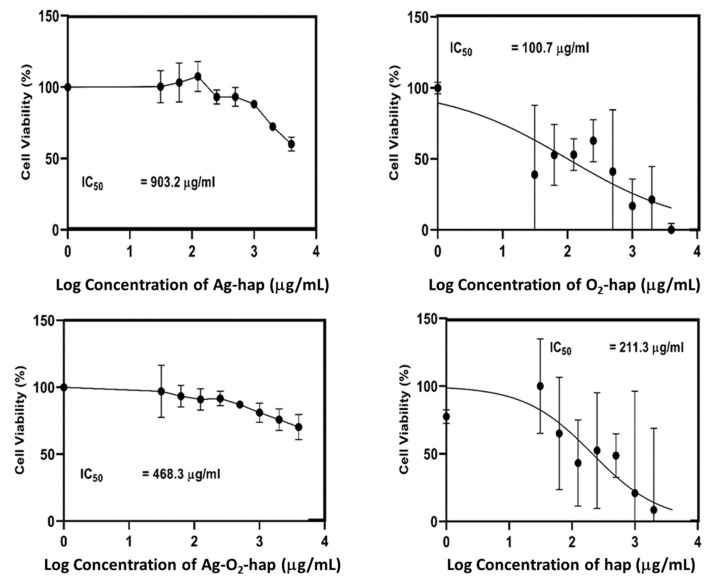
IC_50_ value determined for each type of apatite sample.

To further investigate the cytotoxicity of the doped apatite compared to the hap reference, a log dose response analysis was conducted to provide information on the half-maximal inhibitory concentration (IC_50_) of each sample. Results of this IC_50_ analysis are shown in Figure 9. The half-maximal inhibitory concentration (IC_50_) of the O_2_-hap was found to be the lowest (100.7 µg/mL). In contrast, the Ag-hap sample showed the highest IC_50_ (903.2 µg/mL), also compared to the other doped apatites. When the peroxide apatite was co-doped with Ag, the IC_50_ increased significantly to 468.3 µg/mL in comparison to the original O_2_-hap, pointing out a decreasing biocompatibility tendency upon Ag incorporation. As a reference, the IC_50_ of the hap reference reached 211.3 µg/mL, thus somewhat higher than for the safest material tested, namely O_2_-hap.

Overall, the four materials were thus found to be non-cytotoxic (Figure 8), and the O_2_-hap appeared as the safest type of apatite tested in terms of IC_50_ (Figure 9). The behavior of osteoblast cells when contacted with the materials was also directly observed by SEM analyses (Figure 4b): the cells showed a significant tendency to spread across the materials surface, with extended filopodia, thus confirming a good cell–material interface.

### 3.3. Processing into 2D Membranes and 3D Scaffolds

All of the above results therefore confirmed the relevance of peroxidated apatites as highly biocompatible and antibacterial compounds for bone applications. In the last part of this work, we aimed at showing the possibility to use them not only in the form of powders or compacted pellets, as is shown in Figure 4a, but also in association with polymer components to form composite organic–inorganic scaffolds with potentially tailorable (porous) networks. One particularly appealing approach to obtain 2D membranes and 3D scaffolds from thermally metastable compounds is freeze-casting, which consists of the low temperature crystallization–sublimation of the polymer solvent. In our case, combination of polymers with peroxidated apatite is expected to lead to polymer-based scaffolds with embedded O_2_-hap particles. A successful example of this approach was reported recently in the case of a PLGA/carbonated apatite composites [53]. In the present work, two freeze-casting strategies were developed, as presented below.

**Figure 10:**
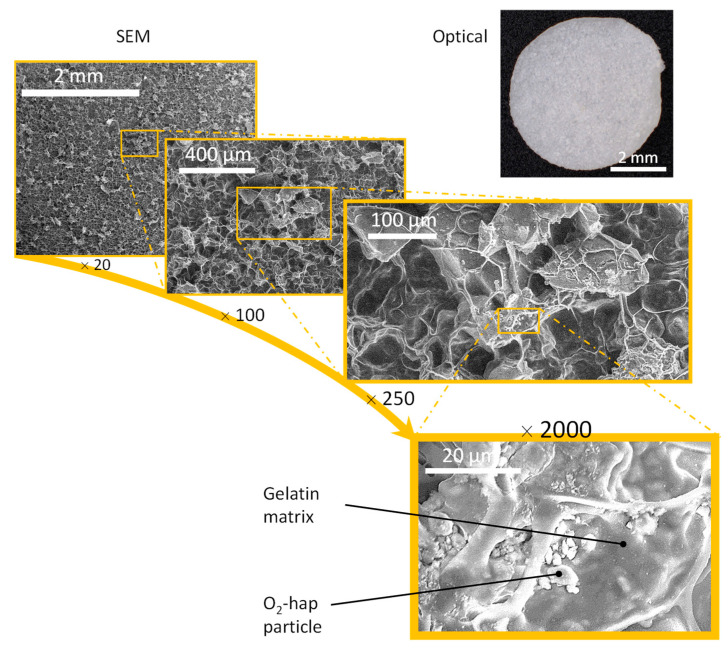
Optical (top right) and SEM observations of 2D inorganic–organic composite membranes obtained by freeze-casting, associating O_2_-hap particles with gelatin.

**Figure 11:**
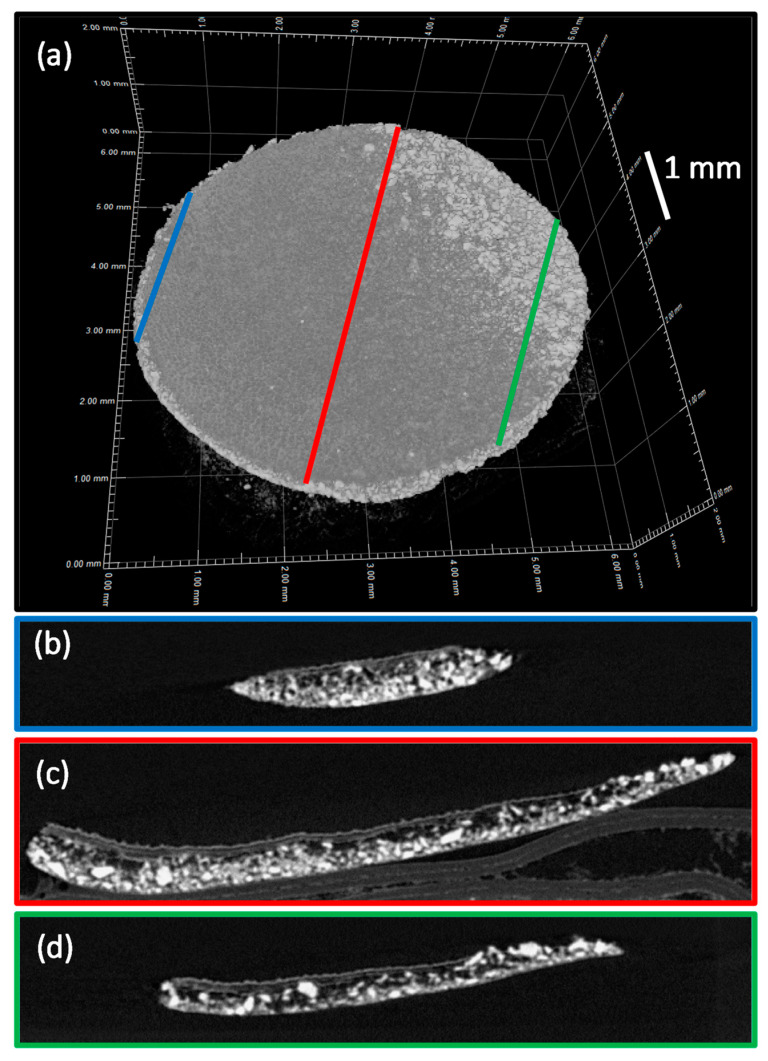
Three-dimensional µCT images (**a**) and µCT sections images ((**b**–**d**), labeled according to the color code of the main image) of 2D inorganic–organic composite membranes obtained by freeze-casting, associating O_2_-hap particles with gelatin.

In our first freeze-casting strategy, we prepared 2D inorganic–organic membranes (6 mm diameter) associating peroxidated apatite and gelatin. The latter consisted of hydrolyzed collagen and may thus provide a highly biocompatible proteic-based network for use in biomedical engineering. Observation by optical and electron microscopies of the membranes obtained (Figure 10) indeed evidenced the formation of a continuous network associated with O_2_-hap particles partly entrapped in the structure. SEM pictures demonstrated that the bottom surface of membranes was homogeneously structured by both porosity of 10–20 μm and aggregates of apatite particles from a few microns to several hundred of microns (Figure 10). The strong agglomeration tendency of apatite particles was confirmed by μCT analysis (Figure 11). Indeed, a segregation was observed between a polymer-rich zone on the top of membranes and a second one with high amounts of agglomerated apatite particles (dense phases appearing as brilliant points on b, c, and d pictures). This phenomenon was enhanced by using apatite powder without further sonication, bigger aggregates leading to sedimentation during the freeze-casting process. Moreover, the porosity observed by SEM was confirmed to be only localized at the surface due to a low amount of water (H_2_O crystals acting as porosity templates). Such a “Janus” membrane is particularly interesting for filling alveolar sockets after tooth extraction to avoid infection via both the intrinsic antibacterial properties of O_2_-hap and the polymer playing a role of physical barrier to prevent microorganism infiltration. They are also expected to provide a favorable environment for underlying bone healing, as roughness associated to apatite agglomerates and surface porosity could favor osteoblast adhesion and proliferation.

**Figure 12:**
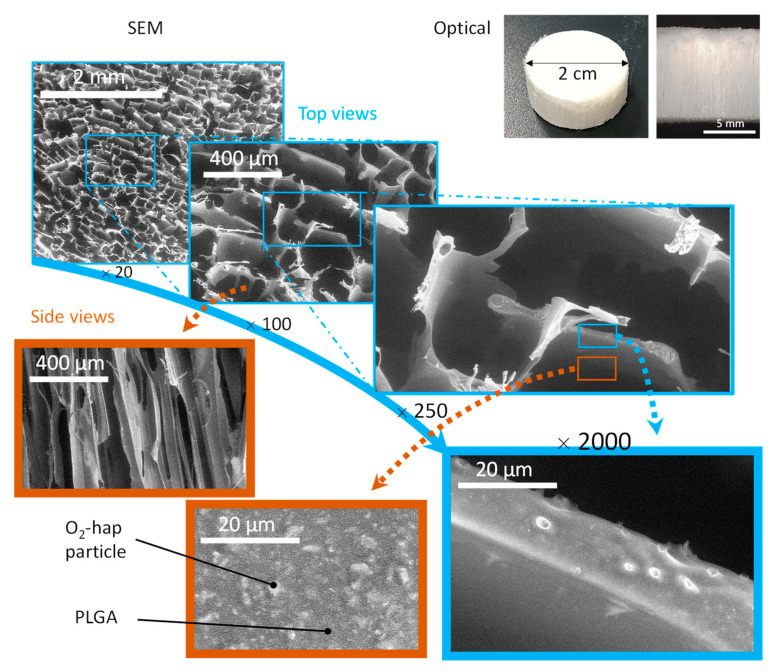
Optical (top right) and SEM observations of 3D inorganic–organic composite scaffolds obtained by freeze-casting, associating O_2_-hap particles with PLGA.

Our second freeze-casting strategy aimed to combine O_2_-hap particles and a PLGA polymer matrix to obtain 3D porous scaffolds (typically, one aims for the final dimensions of 2 cm diameter, 1 cm height for a mass of ca. 200 mg). PLGA is a biodegradable polymer widely used in medical devices [54,55], including in combination with apatite. Preliminary experiments with carbonated apatite pointed out the relevance of PLGA/apatite formulations for freeze-casting [53], typically using dimethyl carbonate (DMC) as solvent. SEM observations of the median section highlight homogeneous anisotropic cellular porosity. The latter is the template of DMC crystals resulting from the unidirectional freezing front progression (Figure 12). Pore section of 200–300 μm fits perfectly with the targeted range for cell colonization and angiogenesis [56]. At higher magnification, SEM pictures demonstrate the homogeneous distribution of apatite particles within the polymer matrix walls. This is of major interest to improve mechanical properties and, in particular, composite rigidity due to the decrease of polymer segment mobility blocked by inorganic fillers [53].

**Figure 13:**
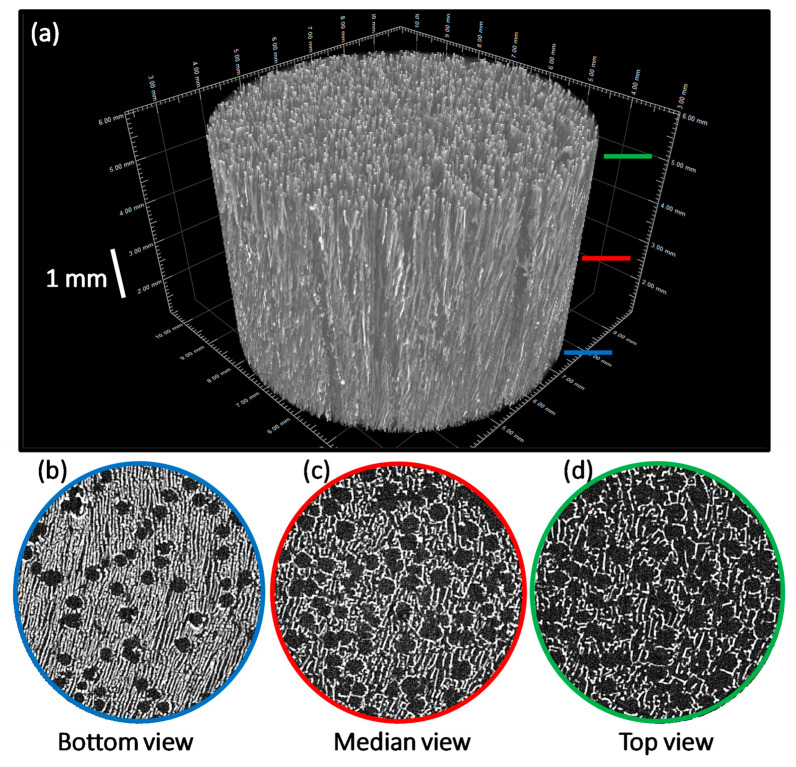
Three-dimensional µCT images (**a**) and µCT sections images ((**b**–**d**), labeled according to the color code of the main image) of 3D inorganic–organic composite scaffolds obtained by freeze-casting, associating O_2_-hap particles with PLGA.

The scaffolds’ porous structure was also analyzed using µCT. The 3D views of the samples (Figure 13a) confirmed the presence of an anisotropic macroporosity oriented along the temperature gradient, as expected with the unidirectional freeze-casting process [57]. Moreover, the whole porous network was interconnected (see the green network, Figure S5, Supporting Information). No preferential zone composed either of polymer or apatite was detected, confirming the good spatial distribution of fillers. Considering cross-sections pictures (Figure 13b–d), two morphologies of porosity could be distinguished, respectively, tubular and cellular [58,59]. The planar structure of DMC molecule preferentially lead to planar crystals but these two kinds of porosity could be explained by a balance between crystal nucleation and growth phenomena. Indeed, the tubular macroporosity was created by crystals merging together, just after the initial nucleation, inducing larger crystals. The cellular porosity resulted from intercrystalline nucleation phenomenon, directly inside the solute-rich phase and by binding tubular pores together. Beyond the shape of pores, a gradient of diameter was also observed. The latter is mainly explained by a high initial solidification rate at the bottom of the mold leading to more favorable crystal nucleation over crystal growth. Once the solidification rate was stabilized, crystals propagation and growth were kinetically favored. In any case, these results demonstrate the presence of oriented and open porosity throughout the scaffold, necessary for cell colonization and vascularization. Such scaffolds (that may be cut subsequently to adapt to existing bone defects) could be considered for bone repair applications in non-load bearing sites, taking into account (i) the high biocompatibility and tailorable resorption rate of PLGA (e.g., via the modulation of the lactic-to-glycolic acid and apatite/polymer ratios), (ii) the bone-forming ability of biomimetic apatite, and (iii) the hemostatic and antibacterial properties of the peroxide doping [60].

**Figure 14:**
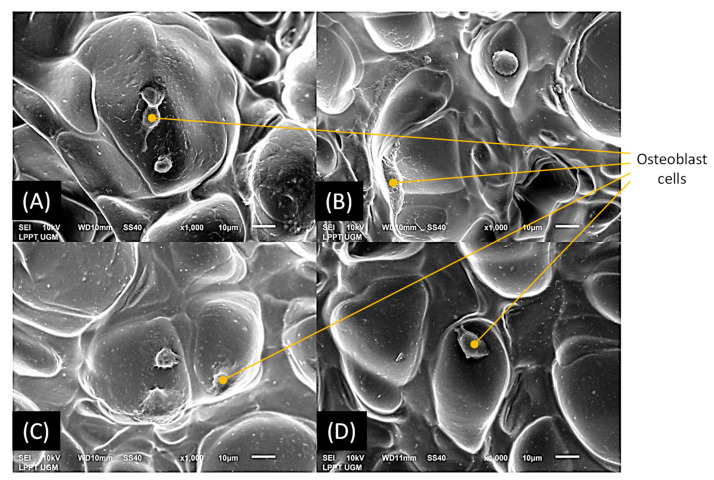
MC3T3E1 osteoblast cells on apatite-gelatin 2D membranes: (**A**) Ag-hap, (**B**) O_2_-hap, (**C**) Ag-O_2_-hap, and (**D**) hap.

In the above section, we showed the possibility to use biomimetic apatites including samples doped with peroxide ions in the fabrication of composite 2D membranes or 3D scaffolds by association with biocompatible polymers, namely gelatin and PLGA. Controlling the formulation (apatite/polymer/solvent ratios, polymer nature) and process parameters (freezing temperature and direction) can allow tuning the final properties and thus the applications of the resulting composite. These polymers have been widely used for biomedical applications, e.g., in bone substitutes elaboration, and were shown to be highly biocompatible. Indeed, seeding osteoblast cells on scaffolds obtained in the present work confirmed that the cells attached well to the material surfaces regardless of the type of apatite, as shown in Figure 14 in the illustrative example of 2D membranes.

## 4. Conclusions

In this work, we developed a safe-by-design type of biomaterial based on biomimetic bone-like apatite associated to oxygenated species, namely peroxide ions O_2_^2−^, close to the ones naturally generated in vivo to fight against pathogenic agents. For comparative purposes, similar compounds doped with Ag^+^ or co-doped with O_2_^2−^ and Ag^+^ were also prepared, along with the non-doped reference biomimetic apatite. We checked the bone-like features of the apatite phases obtained and explored their biological behavior by way of several complementary tests. All doped apatites proved to be significantly antibacterial toward four major pathogenic bacteria involved in periodontitis and more generally bone infection. The samples also proved to be non-cytotoxic to osteoblast cells. In particular, peroxidated apatite exhibited a very low IC_50_ value toward an osteoblast, even lower than raw undoped apatite. We then demonstrated the possibility to associate such doped apatite with two biocompatible polymers, gelatin and PLGA, to prepared composite 2D membranes and 3D scaffolds of relevance to the bone regeneration field. Such bio-inspired highly biocompatible, antibacterial compounds are promising candidates for bone tissue engineering.

## Figures and Tables

**Table 1:** Samples description and synthesis conditions.

Sample Ref	% H_2_O_2_ Initial (vol.%)	Maturation Time (Days)	Final Contents (mol Per Unit Cell Formula *)		Selected for Biological Tests
			Ag^+^	O_2_^2-^	
O_2_-hap-10-1	10	1	0	0.39	X (denoted “O_2_-hap”)
O_2_-hap-25-1	25	1	0	0.43	
O_2_-hap-50-1	50	1	0	0.47	
O_2_-hap-10-3	10	3	0	0.42	
O_2_-hap-25-3	25	3	0	0.47	
O_2_-hap-50-3	50	3	0	0.51	
O_2_-hap-10-7	10	7	0	0.46	
O_2_-hap-25-7	25	7	0	0.53	
O_2_-hap-50-7	50	7	0	0.59	
Ag-hap-0-1	0	1	0.11	0	X (denoted “Ag-hap”)
Ag-O_2_-hap-10-1	10	1	0.14	0.41	X (denoted “Ag-O_2_-hap”)
Ag-O_2_-hap-25-1	25	1	0.15	0.53	
Ag-O_2_-hap-50-1	50	1	0.16	0.46	
Ag-O_2_-hap-10-3	10	3	0.15	0.46	
Ag-O_2_-hap-25-3	25	3	0.15	0.50	
Ag-O_2_-hap-50-3	50	3	0.16	0.56	
Ag-O_2_-hap-10-7	10	7	0.16	0.52	
Ag-O_2_-hap-25-7	25	7	0.16	0.59	
Ag-O_2_-hap-50-7	50	7	0.16	0.66	
hap-0-1	0	1	0	0	X (denoted “hap”)

* assessed by assuming 6 phosphate groups per apatite unit cell.

## Data Availability

Data are available by contacting the corresponding author(s).

## Supplementary Materials

The following supporting information can be downloaded at: https://www.mdpi.com/article/10.3390/jfb13030144/s1: Figure S1: Additional XRD patterns, obtained for apatite compounds prepared under varying conditions in terms of (top graph) H_2_O_2_ initial amounts in the precipitating medium and (bottom graph) apatite maturation times. The reference names refer to Table 1 from the main text. Figure S2: FTIR spectra for apatite compounds prepared under increasing initial amounts of H_2_O_2_ for an apatite maturation time of 1 day. The second graph is a zoomed view on the 425–1500 cm^−1^ domain. The main phosphate band attributions have been added, with reference to bone-like apatite. Figure S3: Replicated results (quadruplicate) of antibacterial tests with P. gingivalis (Pg), A. actinomycetemcomitans (Aa), F. nucleatum (Fn), and S. aureus (Sa) for all four types of apatite samples: Ag-hap (A), O_2_-doped (B), Ag-O_2_-hap (C), and hap (D). Figure S4: SEM (a) and EDX (b) analyses of the four types of apatite starting powders, in secondary electron (SE) and backscattered electron (BSE) modes. Initial magnification of ×1000. Figure S5: Visualization of the interconnected porous network (green) obtained thanks to the analysis of μCT results (Vg Studio Max software). Tables S1 to S7: Results of bacterial growth inhibition of the four types of hap (ref) and doped hap, and summary of one-way ANOVA analyses.
