# Supplementary material for: Safe-by-Design Antibacterial Peroxide-Substituted Biomimetic Apatites: Proof of Concept in Tropical Dentistry
Source: J Funct Biomater. 2022 Sep 7;13(3):144. doi: 10.3390/jfb13030144 (PMC9503752; doi:10.3390/jfb13030144)
Supplement: Supplementary file 1 [file jfb-13-00144-s001.zip › jfb-1852287-supplementary.pdf]

Supporting Information

Figure S1: XRD patterns for apatite compounds prepared under varying conditions in terms of (top graph) H_2O_2 initial amounts in the precipitating medium and (bottom graph) apatite maturation times. The reference names refer to **Table 1** form the main text.

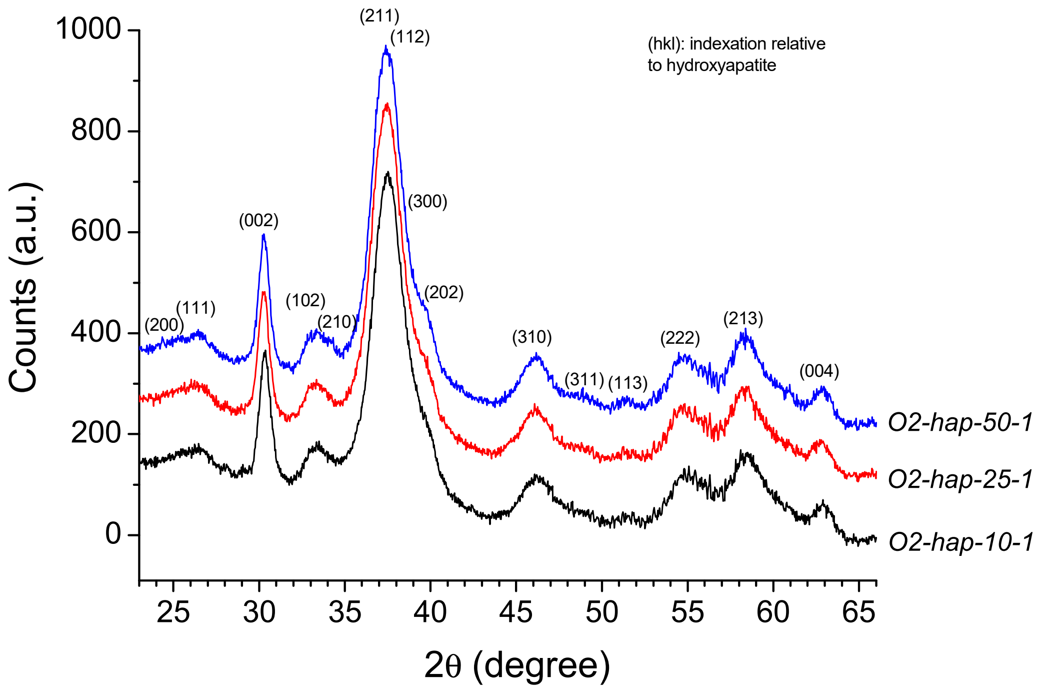

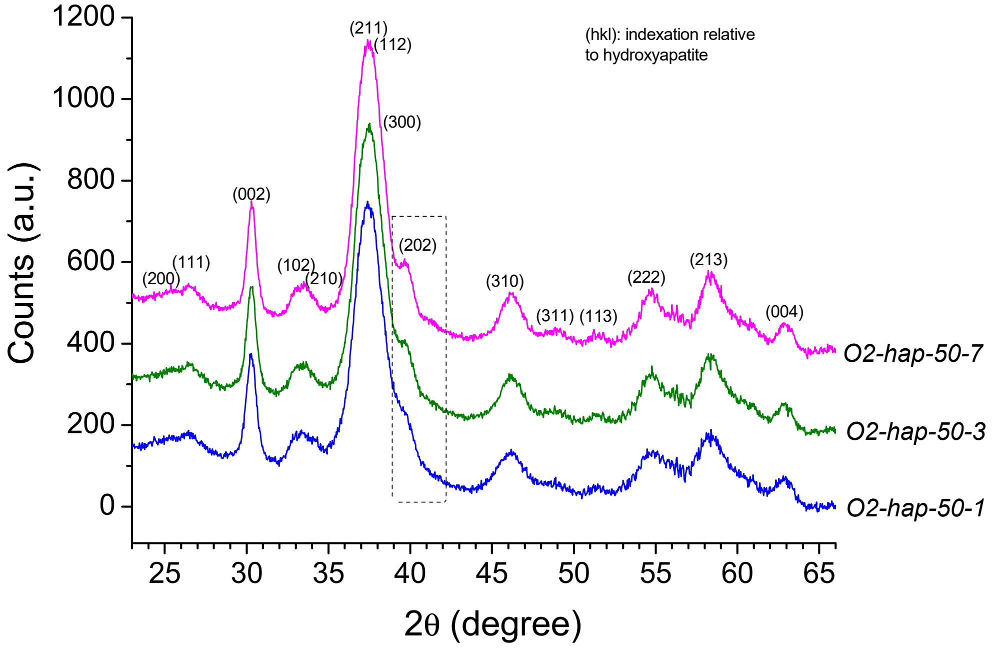

Figure S2: FTIR spectra for apatite compounds prepared under increasing initial amounts of H_2O_2 for an apatite maturation time of 1 day. The second graph is a zoomed view on the 425-1500 cm^{-1} domain. The main phosphate band attributions have been added, with reference to bone-like apatite.

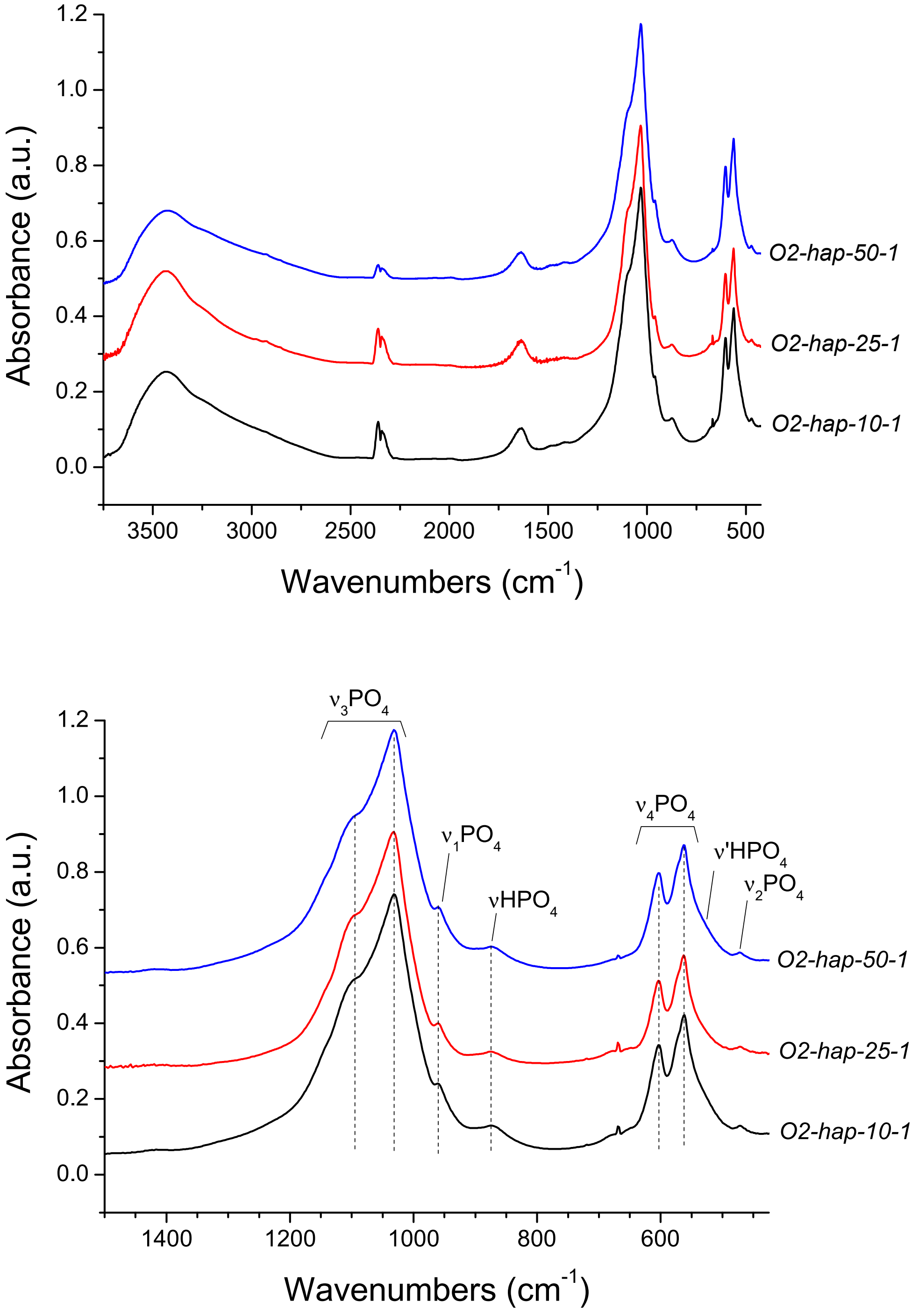

Figure S3: Replicated results (quadruplicate) of antibacterial tests with *P. gingivalis* (Pg), *A. actinomycetemcomitans* (Aa), *F. nucleatum* (Fn) and *S. aureus* (Sa)for all 4 types of apatite samples: Ag-hap (A), O2-doped (B), Ag-O2-hap (C) and hap (D).

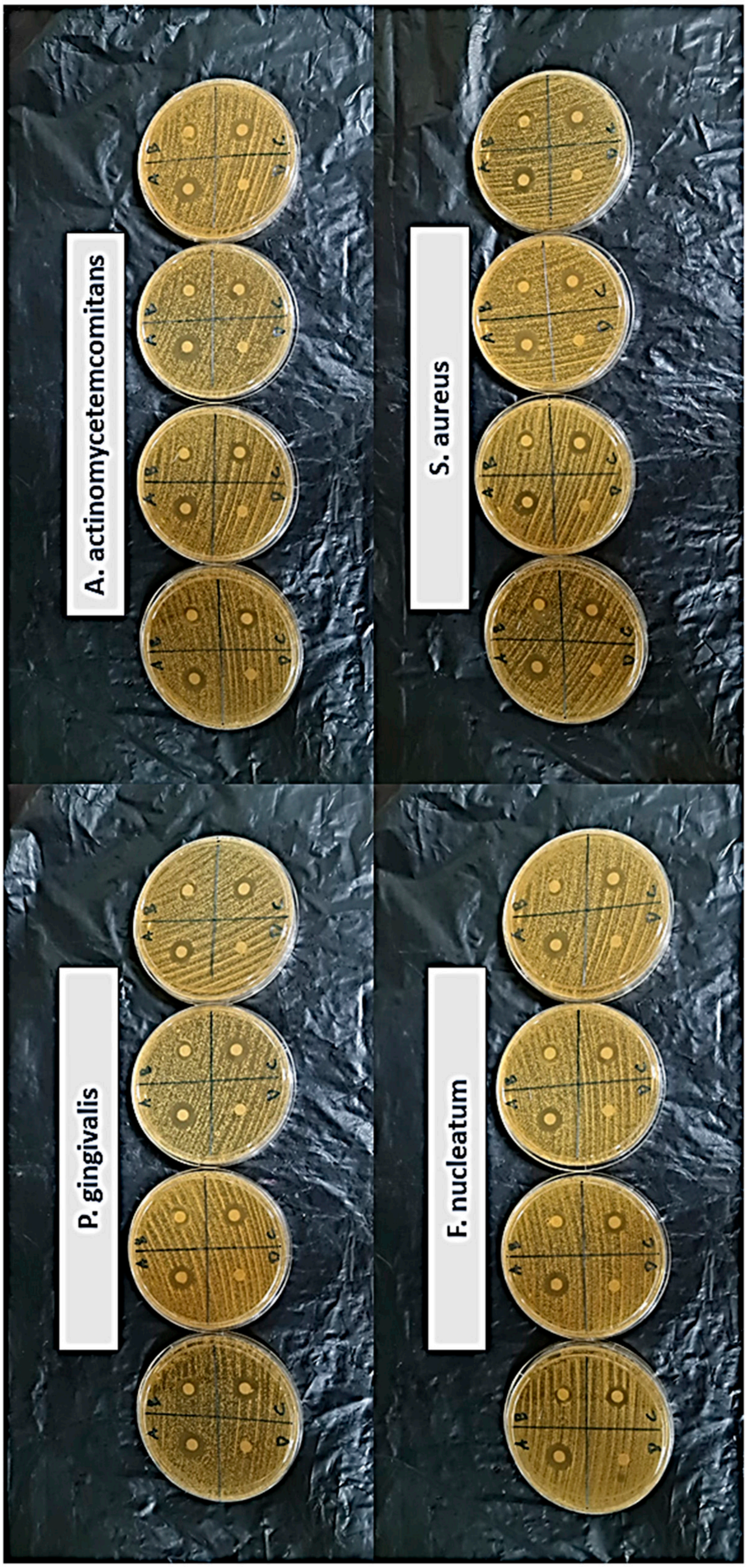

Figure S4: SEM (a) and EDX (b) analyses of the 4 types of apatite starting powders, in secondary electron (SE) and backscattered electron (BSE) modes. Initial magnification of x1000.

(a)

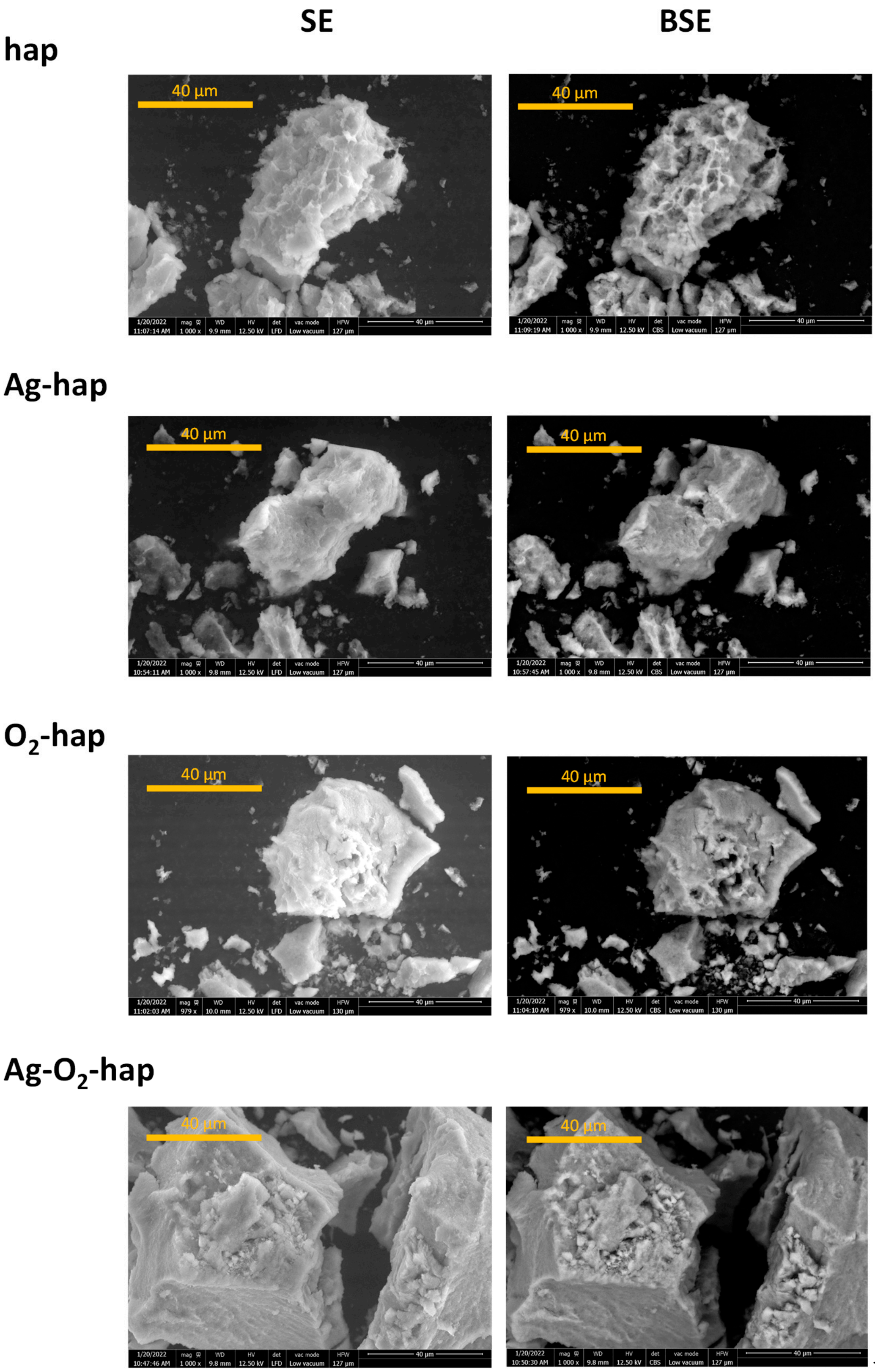

--/→

(b)

hap

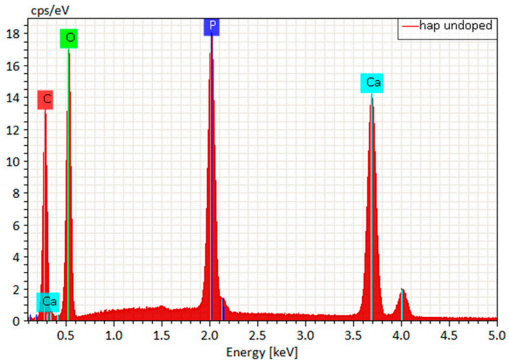

Ag-hap

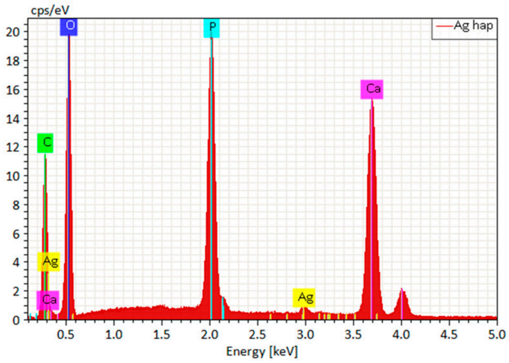

O_2-hap

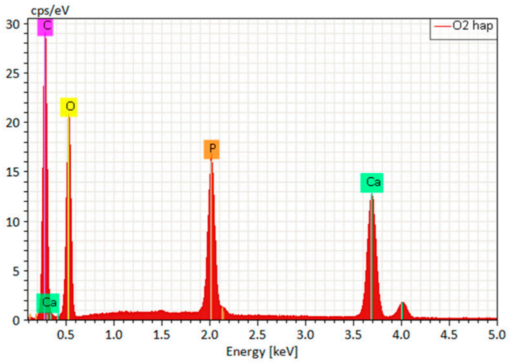

Ag-O_2-hap

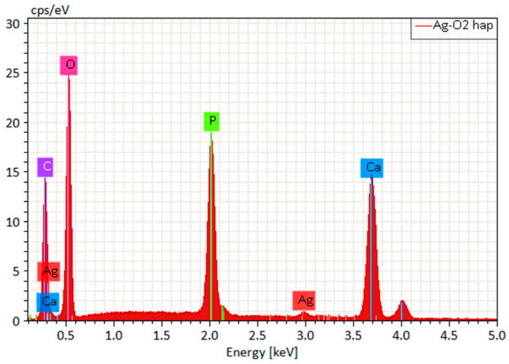

Figure S5: Visualization of the interconnected porous network (green) obtained thanks to the analysis of µCT results (Vg Studio Max software)

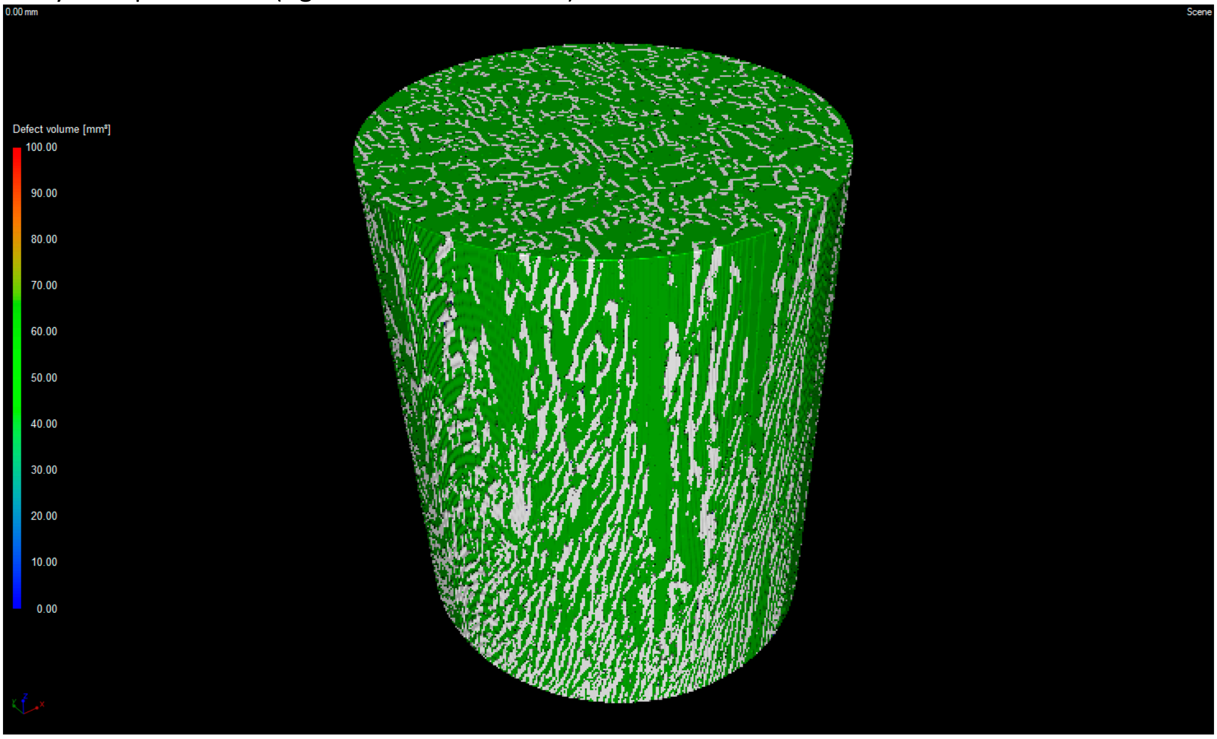

Table S1: Results of bacterial growth inhibition of the 4 types of hap (ref) and doped hap.

Types of Apatite	***Porphyromonas gingivalis***	***Aggregatibacter actinomycetem-comitans***	***Fusobacterium nucleatum***	***Staphylococcus aureus***
Ag-hap (A)	12.900±0.53	12.875±0.17	11.950±0.11	14.875±0.38
O_2-hap (B)	8.833±0.19	8.125±0.12	7.650±0.12	9.100±0.25
Ag-O_2-hap (C)	11.050±0.30	10.437±0.26	9.575±0.44	12.675±0.12
hap (D)	0.000	0.000	0.000	0.000

Table S2: Summary of one-way ANOVA on the antimicrobial properties of the samples against *P. gingivalis*.

**Sample**	**Mean**	**Std Deviation**	**Std. Error**	***p***
Ag-hap	12.9000	0.5292	0.2646	0.001*
O_2-hap	8.3250	0.1848	0.0924	
Ag-O_2-hap	11.0500	0.3000	0.1500	

*Significance p<0.05

Table S3: Summary of one-way ANOVA on the antimicrobial properties of the samples against *A. actinomycetemcomitans*.

**Sample**	**Mean**	**Std Deviation**	**Std. Error**	***p***
Ag-hap	12.8250	0.1658	0.0829	0.001*
O_2-hap	8.1250	0.1190	0.0595	
Ag-O_2-hap	10.4375	0.2562	0.1281	

*Significance p<0.05

Table S4: Summary of one-way ANOVA on the antimicrobial properties of the samples against *F. nucleatum*.

**Sample**	**Mean**	**Std Deviation**	**Std. Error**	***p***
Ag-hap	11.9500	0.1080	0.0540	0.001*
O_2-hap	7.6500	0.1915	0.0957	
Ag-O_2-hap	9.5750	0.4406	0.2203	

*Significance p<0.05

Table S5: Summary of one-way ANOVA on the antimicrobial properties of the samples against *S. aureus*.

Sample	Mean	Std Deviation	Std. Error	*p*
Ag-hap	14.1875	0.3750	0.1875	0.001*
O_2-hap	9.1000	0.2483	0.1242	
Ag-O_2-hap	12.6750	0.1190	0.0595	

*Significance p<0.05

Table S6: Summary of one-way ANOVA on the protein adsorption.

Sample	Mean	Std Deviation	Std. Error	*p*
Ag-hap	0.9197	0.0625	0.0361	0.001*
O_2-hap	0.4797	0.1333	0.0770	
Ag-O_2-hap	0.2570	0.0702	0.0405	
hap	0.593781	0.19631483	0.113342	

*Significance p<0.05

Table S7: Summary of one-way ANOVA on MC3T3E1 osteoblast cell adhesion.

Sample	Mean	Std Deviation	Std. Error	*p*
Ag-hap	99.3233	3.4900	2.0150	0.001*
O_2-hap	105.6233	5.2594	3.0365	
Ag-O_2-hap	65.7533	1.0001	0.5774	
hap	72.7966	5.5524	3.2057	

*significance p<0.05
